# The Bacterial Cell Wall Components Lipopolysaccharide and Peptidoglycan Initiate Divergent Local Tissue and Systemic Inflammatory Response Profiles in the Chicken Model

**DOI:** 10.3390/ani14243661

**Published:** 2024-12-18

**Authors:** Kristen A. Byrne, Gisela F. Erf

**Affiliations:** Department of Poultry Science, Center of Excellence for Poultry Science, University of Arkansas System Division of Agriculture, Fayetteville, AR 72701, USA; kristen.byrne@usda.gov

**Keywords:** inflammatory response, lipopolysaccharide, peptidoglycan, chicken, leukocyte, lymphocytes, cytokines, innate immunity, microbe associated molecular patterns

## Abstract

The innate immune system plays an important role in the defense against pathogens, whereby the ability to rapidly mount an effective inflammatory response is critical in the elimination/containment of the infection. To better understand the nature of the inflammatory responses to bacterial components in chickens, we used the growing feather (GF) cutaneous bioassay together with blood sampling to examine the local and systemic inflammatory responses initiated by intradermal (i.d.) GF-pulp injection of lipopolysaccharide (LPS) from *Salmonella* Typhimurium or peptidoglycan (PGN) from *Staphylococcus aureus.* Three studies were conducted in egg-type chickens between 9 and 15 weeks of age; Study 1 and 2 examined the leukocyte response profiles to a 100-fold dose range of LPS or PGN over 24 h or 7 d, respectively; Study 3 examined the leukocyte- and cytokine mRNA-profiles in pulps in response to LPS and PGN concurrently over 72 h. I.d. injection of LPS stimulated a heterophil and monocyte/macrophage dominated response in both GF-pulps and blood that was resolved by 48–72 h and differed based on dose administered. The inflammatory response stimulated by PGN was characterized by rapid infiltration of lymphocytes in GF-pulps with sustained high levels of T and B cells over 5–7 d and was neither affected by PGN dose nor reflected in the blood. Limited cytokine transcriptome analyses did not reveal differences that could explain the divergent response profiles to LPS versus PGN. More research is needed to understand the mechanisms underlying the divergent inflammatory responses to LPS and PGN in chickens.

## Simple Summary

The innate immune system plays an important role in the early defense against pathogens. To better understand the chicken’s innate inflammatory responses, we concurrently examined the cellular responses at the injection site and in the blood following intradermal (i.d.) injections of lipopolysaccharide (LPS) or peptidoglycan (PGN) into the pulp of growing feathers (GFs). Both LPS from Gram-negative bacteria and PGN from Gram-positive bacteria are potent stimulators of innate inflammatory responses. The time-course study conducted revealed similarly elevated levels of heterophils and macrophages in injected GF-pulps during the first 24 h for both LPS and PGN. However, PGN also stimulated rapid infiltration of lymphocytes, with high levels sustained for 7 days post-injection. The i.d. injection of LPS also affected leukocyte profiles in the blood, whereas PGN did not. Overall, the temporal, qualitative, and quantitative differences in the inflammatory responses to LPS and PGN suggest different innate immune response mechanisms in the defense against infection with Gram-negative and Gram-positive bacteria in chickens.

## 1. Introduction

With the push to reduce antibiotic use in animal agriculture, new approaches are needed to manage poultry health, including production of animals with robust innate immune system defenses. The innate immune system has evolved to sense and respond to microbial invasion by recognition of molecular features common to groups of microbes known as microbe associated molecular patterns (MAMPs). Recognition of MAMPs is achieved by cell associated or soluble pattern recognition receptors (PRRs) that are specific to microbial components critical for the survival of the microbe and foreign to the host [1,2]. Moreover, cell associated PRRs are strategically located to sense microbes where they are most likely encountered by cells, e.g., on the plasma membrane, the endosome, or cytosol. The binding of MAMPs by PPRs then activates a rapid and appropriate response to eliminate the microbial invader. Common bacterial MAMPs recognized by PRRs of innate immune cells are lipopolysaccharide (LPS), the outer membrane component of Gram-negative bacteria, and peptidoglycan (PGN), a structural component of all bacteria that is most abundant in Gram-positive bacteria [3].

In both avian and mammalian species, recognition of LPS through toll like receptor (TLR) 4 on tissue resident leukocytes, such as macrophages, induces an inflammatory response characterized by chemokine and cytokine induced vascular changes and rapid recruitment of phagocytes from the blood to the infected tissue [3,4,5,6,7]. Recently, both local tissue and systemic (blood) acute inflammatory response profiles to intradermal injection of LPS were established in broilers using a two-window approach [7]. Specifically, the growing feather (GF) cutaneous test system [8] was used to monitor leukocyte infiltration and activities in GFs before and after intradermal pulp injection of LPS, along with concurrent changes in the proportions and concentrations of peripheral blood leukocytes [7]. This minimally invasive “two-window” approach further confirmed the similarities in the acute inflammatory response initiated by LPS in mammals and chickens and established a comprehensive approach to the study of local cellular–/tissue– and systemic–immune system responses in an avian model.

Recognition of PGN by the innate immune system is important as PGN is common in all species of bacteria and, as such, is recognized by multiple PRRs, including TLR 2 [3,9,10,11], nucleotide-binding oligomerization domains 1 and 2 (NOD1 and NOD2), and various peptidoglycan recognition proteins [1,12,13]. In vitro research with chicken peripheral blood heterophil and macrophage cell lines indicated that transcriptional expression of the inflammatory genes IL1B, IL6, and CXCL8 is upregulated upon exposure to PGN [14,15]. Data on in vivo inflammatory responses initiated by PGN are, however, limited. Indirect evidence of PGN’s inflammatory activity in vivo was established in time-course studies evaluating leukocyte infiltration profiles in chickens in response to intradermal injection of Gram-positive bacteria (*Mycobacterium butyricum*) into wattles and wing webs over 3 days, and in GF-pulps over 7 days. While a transient increase in heterophils was observed within 6 h post-injection (p.i.), the response was dominated by sustained infiltration of mononuclear cells, with T and B lymphocytes reaching peak levels at 2 d and macrophages at 3 d before returning to near pre-injection levels by 7 d p.i. [8].

The objective of this study was to use the dual-window approach to further define the local (pulp) and systemic (blood) inflammatory responses to intradermal pulp injection of LPS, as well as to conduct novel, comprehensive analyses of these in vivo responses to PGN in the chicken model. For both LPS and PGN, a dose response over a 100-fold range was conducted, and GFs and blood were sampled more frequently, including earlier (2, 4, and 8 h p.i.) and later (up to 7 d p.i.) time points, compared to a previous LPS study [7]. Ex vivo sample analyses included monitoring leukocyte infiltration (heterophils, macrophages, B cells, and T cell subsets) and relative cytokine/chemokine mRNA expression in injected GF-pulps, along with changes in the concentrations in blood leukocytes.

## 2. Materials and Methods

### 2.1. Experimental Animals

Non-vaccinated, layer-type chickens from the Light-brown Leghorn and Massachusetts Brown-line breeding populations maintained by G. F. Erf at the University of Arkansas System Division of Agriculture (UADA) Poultry Research Farm, Fayetteville, AR, were raised in HEPA filtered rooms in the UADA Poultry Health Laboratory, Fayetteville, AR in floor pens (0.9 m × 1.8 m) on wood shaving litter. For Study 1 and 2, equal numbers of males and females were kept in 4 pens, 10 birds/pen; for Study 3, males were kept in 3 pens, 7 birds/pen. Food and water were available ad libitum, and standard light and temperature protocols were followed [16]. All procedures involving animals were approved by the University of Arkansas Institutional Animal Care and Use Committee (approval #15021).

### 2.2. Intradermal Injection and Collection of Growing Feathers

Preparation and intradermal pulp injection of growing feathers (GFs) were conducted as described in [7]. Briefly, to ensure availability of uniform GFs for intradermal pulp injection, 10–15 GFs at various stages of development were plucked in a row from each breast tract of a chicken and allowed to regenerate for 18 days. For injection, the emerging barbs and portion of the sheath above the pulp epidermis were cut off with scissors. Using syringes with 0.01 mL gradation and 31-gauge × 8 mm needles (BD, Franklin Lakes, NJ, USA). The center of the pulp dermis of the GFs was injected with 10 μL of test-material in endotoxin-free Dulbecco’s phosphate-buffered saline (PBS; Sigma, St. Louis, MO, USA). For each chicken, GFs were collected before injection and at various times post-injection (p.i.) for ex vivo laboratory analyses. Collected GFs were placed into tubes containing cold PBS and kept on ice until same-day preparation and direct immunofluorescent staining of pulp cell suspensions for leukocyte population analysis by flow cytometry. For RNA isolation, collected GFs were also placed in a room temperature RNA preservation buffer (RNAlater^®^ Thermo Fisher, Waltham, MA, USA). Following a gradual decrease in temperature as described in the RNAlater^®^ protocol manual, samples were stored at −20 °C until RNA isolation and processing for cytokine expression analyses by RT-qPCR.

### 2.3. Blood Collection

Heparinized syringes (3 mL) with 25-gauge × 1-inch needles were used to collect 0.5 to 1 mL of blood from the wing veins of each bird at each GF collection time point. Blood samples were used for same-day blood cell profile analysis via automated hematology (Cell-Dyn, Abbott Diagnostics, Abbott Park, IL, USA).

### 2.4. Preparation of Pulp Cell Suspensions, Immunofluorescent Staining, and Cell Population Analyses by Flow Cytometry

Feather pulp suspensions were prepared on the day of collection as described in [8]. Briefly, the living tissue (pulp) was removed from the GF and then incubated in 0.1% collagenase/dispase (Collagenase type IV, Life Technologies, Carlsbad, CA, USA; Dispase II, Sigma-Aldrich, St. Louis, MO, USA) at 40 °C for 15 min and pushed through a 60 µm nylon mesh to prepare single-cell suspensions. Pulp cell suspensions were washed and resuspended in PBS+ buffer (PBS, 1% bovine serum albumin and 0.1% sodium azide; VWR, Radnor, PA, USA) for immunofluorescent staining with a panel of fluorescently labeled (FITC, PE, or SPRD) mouse monoclonal antibodies (Southern Biotechnology Associates, Inc., Birmingham, AL, USA) using a three-color direct staining procedure. Specifically, all suspensions were labeled with a pan leukocyte marker (CD45-SPRD) to identify and gate around all leukocytes before determining individual leukocyte subpopulations. Leukocyte subpopulations determined included macrophages (KUL01-PE^+^), B cells (Bu-1-FITC^+^), CD4^+^ T cells (CT4-FITC^+^), CD8α^+^ lymphocytes (CT8-PE^+^), γδ T cells (T cell receptor (TCR)1-PE^+^), αβ1 T cells (TCR2-FITC^+^), and αβ2 T cells (TCR3-PE^+^). Total lymphocyte populations were determined by the addition of the percentage estimates of B cells, γδ T cells, αβ1 T cells, and αβ2 T cells for each cell suspension. Due to the lack of chicken heterophil-specific antibodies, heterophil populations in pulp cell suspensions were determined based on size (FSC) and granularity (SSC) characteristics of CD45^+^ leukocytes, as described in [17]. A pool of all cell suspensions was incubated with a cocktail of FITC, PE, and SPRD labeled mouse IgG1 isotype controls to confirm absence of non-specific binding of labeled antibodies and to set the cut-off between fluorescence positive and negative populations. Pooled cell suspensions were also single stained with either CD45-FITC, CD45-PE, or CD45-SPRD to set compensation for three-color analysis. Cell population analysis was conducted using a Becton Dickinson FACSort flow cytometer equipped with a 488-nm argon laser (BD Immunocytometry Systems, San Jose, CA, USA) and the percentage of each leukocyte population in the pulp cell suspensions was determined using CellQuest software 3.2.1. (BD, Franklin Lakes, NJ, USA). For each type of leukocyte, data were expressed as % of total pulp cells.

### 2.5. RNA Isolation, Quantification, and cDNA Synthesis

Pulp tissue from frozen GFs stored in RNAlater^®^ (Ambion, Waltham, MA, USA) was removed as described above. GF-pulps were then homogenized by Tissue Tearor™ (BioSpec Products, Inc, Bartlesville, OK, USA, Model: 985370-395) in a lysis buffer provided in the Qiagen RNeasy^®^ Mini kit (Qiagen Inc., Valencia, CA, USA); total RNA was isolated from homogenates using the same kit. Following the procedure described in [16], RNA integrity and concentration were determined, and RNA (1.5 μg/sample) was transcribed to cDNA using a High Capacity cDNA reverse transcription kit according to the manufacturer’s protocol (Applied Biosystems, Foster City, CA, USA).

### 2.6. Relative Expression of Cytokines

Target gene primers and probes used in this study are listed in Table 1. Primer efficiency and linear amplification range were determined using serial dilutions of a cDNA mix. Real-time quantitative PCR (RT-qPCR) was performed according to French et al. [7]. Relative mRNA expression changes of interleukin (IL) 1 beta (IL1B), IL4, IL6, IL8 (CXCL8), IL10, IL12A, IL21, LPS induced TNF factor (LITAF), and interferon (IFN) alpha (IFNA), beta (IFNB), and gamma (IFNG) genes were normalized against 28S rRNA expression for each sample. Delta Ct values were subtracted from the total number of cycles (40) and data were expressed as (40 − ∆Ct) [18,19].

### 2.7. Blood Profile Analysis via Automated Hematology (CellDyn)

Blood cell concentrations for total WBC, heterophils, monocytes, lymphocytes, basophils, eosinophils, and thrombocytes were determined using an automated hematology analyzer (Cell-Dyn; Abbott Diagnostics, Abbott Park, IL, USA) optimized for chicken blood analyses [7].

### 2.8. Study 1: Local and Systemic Inflammatory Responses to Intradermal Pulp Injection of Various Dosages of Lipopolysaccharide (LPS)

Sixteen, 9-wk-old chickens, four per floor pen, were randomly assigned to one of four treatment groups, two males and two females per treatment. Treatments consisted of i.d. injections of 10 μL of PBS or 1, 10, or 100 µg/mL of LPS from *Salmonella* Typhimurium (Sigma, St. Louis, MO, USA) into the pulps of 20 GFs/bird (10 GFs/breast tract), resulting in 0, 0.01, 0.1, and 1 μg LPS per GF and the designation of treatment groups as PBS, LPS 0.01, LPS 0.1, and LPS 1, respectively. Two injected GFs were collected from each chicken before injection (0 h) and at 2, 4, 8, and 24 h p.i. Collected GFs were placed in cold PBS until preparation, immunofluorescent staining, and leukocyte population analysis by flow cytometry. At each GF collection, heparinized blood was collected for blood cell population analysis.

### 2.9. Study 2: Local and Systemic Inflammatory Responses to Intradermal Pulp Injection of Various Dosages of Peptidoglycan (PGN)

Sixteen, 12-wk-old chickens, four per floor pen, were randomly assigned to one of four treatment groups, with two males and two females per treatment. Treatments consisted of i.d. injections of 10 µL PBS or 5, 50, or 500 µg/mL of PGN from *Staphylococcus aureus* (Invivogen; San Diego, CA, USA) into the pulps of 20 GFs/bird (10 GFs/breast tract) resulting in 0, 0.05, 0.5, and 5 μg PGN per GF and the designation of the treatment groups as PBS, PGN 0.05, PGN 0.5, and PGN 5, respectively. Two injected GF were collected from each chicken before injection (0 h) and at 4 and 8 h and 1, 2, 3, 5, and 7 d p.i. Collected GFs were placed in cold PBS until preparation, immunofluorescent staining, and leukocyte population analysis by flow cytometry. At each GF collection, heparinized blood was collected for blood cell population analysis.

### 2.10. Study 3: Local Leukocyte Infiltration and Cytokine Gene Expression in Response to Intradermal Pulp Injection of PBS, LPS, or PGN

Eighteen, 15-wk-old male chickens, six per floor pen, were randomly assigned to one of three treatment groups, six chickens per treatment. Based on guidance from the dose response studies (Study 1 and 2), treatments consisted of i.d. pulp injections of 20 GFs per chicken (10 GFs/breast tract) with 10 µL PBS or 10 μL of 100 µg/mL of LPS or PGN, resulting in 0 (PBS), 1 μg LPS, or 1 μg PGN per GF. Two injected GFs were collected from each chicken before injection (0 h) and at 4, 8, 24, 48, and 72 h p.i. At each time point, one collected GF was placed into cold PBS until preparation, immunofluorescent staining, and leukocyte population analysis of pulp cell suspensions by flow cytometry; the other GF was stored in RNAlater^®^ until processing and cytokine RNA expression analysis by RT-qPCR.

### 2.11. Statistical Analysis

The experimental unit is the individual chicken with 4 to 6 chickens per treatment. Data were analyzed using Sigma Plot 14.5 Statistical Software (Systat Software, Inc., San Jose, CA, USA). Three-way Analysis of Variance (ANOVA) revealed no sex difference for any of the parameters examined in Study 1 and 2. Hence, for Study 1 and Study 2, data for males and females were pooled for further analysis. For all three studies, a two-way ANOVA or two-way Repeated Measures (RM) ANOVA was conducted to determine significant effects of treatment, time, and treatment-by-time interaction for GF and blood data, respectively. Multiple means comparisons using the Holm–Sidak method were conducted for main effects of time and treatment when no significant interactions were found; when significant interactions were determined, the multiple means comparisons were performed to determine time differences within a treatment group and treatment differences within a time point. For all analyses, differences were considered significant at *p* ≤ 0.05.

## 3. Results

### 3.1. Study 1: Local (GF-Pulp) and Systemic (Blood) Inflammatory Responses to Intradermal Pulp Injection of Various Dosages of Lipopolysaccharide (LPS)

**Figure 1:**
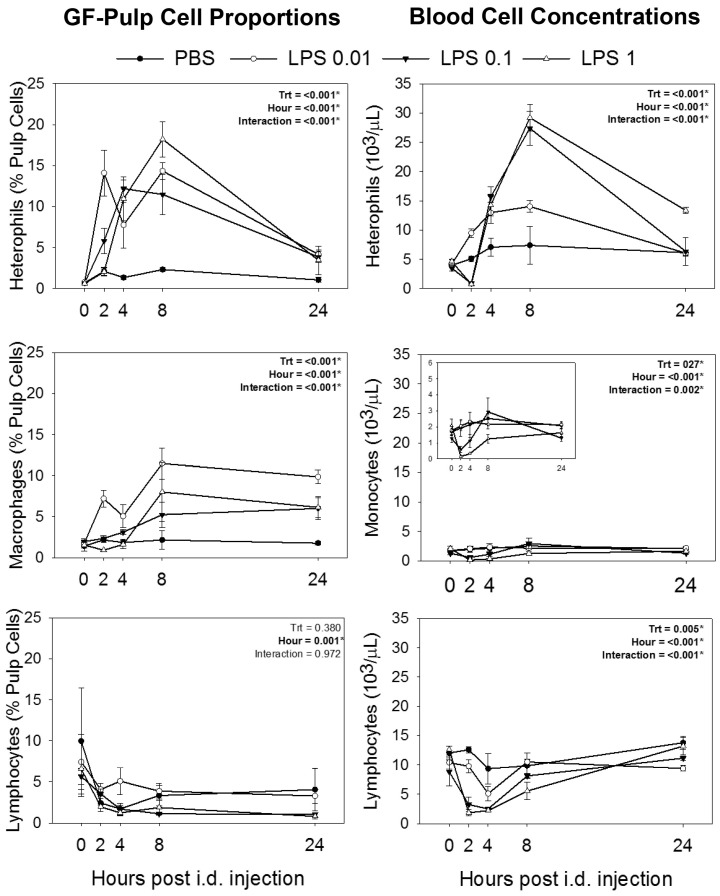
Changes in blood cell concentrations and pulp cell proportions of heterophils, monocytes/macrophages, and total lymphocytes after intradermal injection of lipopolysaccharide (LPS) or endotoxin-free PBS vehicle into the pulp of growing feathers. Twenty growing feathers (GFs) of sixteen, 9-wk-old male and female chickens were injected with PBS (vehicle control), 0.01, 0.1, or 1 μg of LPS per GF. Heparinized blood (0.5–1 mL) and one injected GF from each chicken were collected at 0 (before injection), 2, 4, 8, and 24 h post-injection for leukocyte population analysis. An automated hematology analyzer (Cell-Dyn) was used to determine heterophil, monocyte, and lymphocyte blood cell concentrations (10^3^ cells/μL). Pulp cell suspensions prepared from GF-pulps were immunofluorescently stained with a panel of fluorescence-conjugated mouse monoclonal antibodies to identify chicken macrophages and lymphocytes. Analysis of cell populations was conducted by flow cytometry, and percentages of heterophils were based on size (FSC) and granularity (SSC) characteristics of leukocytes (CD45^+^). Data shown are means ± SEM; * indicates significant effects; n = 4 per time point and treatment group.

Apart from lymphocytes and their subpopulations in GF-pulps, statistical analyses revealed treatment-by-time interactions for heterophil and macrophage proportions (% pulp cells) in GF-pulps, as well as for concentrations of heterophil, monocyte, and lymphocytes in the peripheral blood, indicating that the different treatments resulted in different response profiles over time. Specifically, i.d. pulp injection of PBS vehicle did not result in changes of heterophil, monocyte/macrophage, and lymphocyte levels in pulps and peripheral blood over the 24-h study (Figure 1). Separate analysis of LPS treatments (LPS 0.01, LPS 0.1, and LPS 1) revealed time effects (*p* < 0.001) for each treatment group. Injection of the lowest LPS dose (LPS 0.01; 0.01 μg/GF) resulted in high levels of heterophil infiltration by 2 h post pulp injection (p.i.) and remained near these elevated levels at 4 and 8 h, before decreasing to above pre-injection levels by 24 h (Figure 1). For LPS 0.1 and LPS 1 dosages, pulp heterophil levels were elevated by 4 h p.i and remained at that level for LPS 0.1 but further increased with LPS 1. By 24 h, pulp heterophil levels returned to near pre-injection levels for both LPS 0.1 and 1. In the blood, heterophil concentrations (10^3^ cells/μL of blood) were also affected by i.d. pulp injection of LPS (time effect *p* < 0.001), with LPS 0.01 resulting in elevated heterophil concentrations at 2 h p.i., reaching maximal levels by 4 and 8 h before returning to pre-injection levels by 24 h. Higher dosages of LPS resulted in a drop (*p* ≤ 0.05) of heterophil concentrations at 2 h, then increased greatly by 4 h, reaching peak levels by 8 h. By 24 h, heterophil concentrations returned to pre-injection levels with LPS 0.1, whereas with LPS 1, concentrations remained elevated, dropping to levels observed at 4 h p.i. (Figure 1).

As with heterophils, separate analysis of LPS treatment effects on monocyte/macrophage levels revealed time effects (*p* < 0.001). I.d. pulp injection of LPS 0.01 resulted in monocyte/macrophage infiltration (*p* ≤ 0.05) by 2 h p.i. with similarly elevated levels at 4 h, and a further increase to maximal levels by 8 h that were maintained at 24 h p.i. For both LPS 0.1 and LPS 1, pulp macrophage levels were elevated (*p* ≤ 0.05) by 8 h p.i and remained at that level at 24 h. In the blood, monocyte concentrations did not change in response to LPS 0.01 but dropped below pre-injection levels at 2 h for both LPS 0.1 and LPS 1 (*p* ≤ 0.05). Monocyte concentrations returned to pre-injection levels by 4 h and 8 h for LPS 0.1 and LPS 1, respectively, and remained at that level thereafter (Figure 1).

For lymphocyte levels in the pulp, there were no treatment-by-time interactions (*p* = 0.972) and no treatment differences (*p* = 0.380), but a main effect of time (*p* < 0.001). Overall, all treatments resulted in a decrease in pulp lymphocytes by 2 h p.i. and levels remained below pre-injection for the 24-h period (Figure 1). Two-way ANOVA of individual lymphocyte subpopulations (B cells and CD4-, CD8-, γδ TCR-, aβ1 TCR-, and αβ2 TCR-defined T cell subsets) revealed no treatment-by-time interactions (*p* ≥ 0.765) and no treatment effects (*p* ≥ 0.261), but a main effect of time (*p* < 0.01) for each of the populations examined. However, multiple means comparisons for time effects did not reveal differences between individual time points for each of the cell types. In the blood, lymphocyte concentrations were below pre-injection levels at 4 h for LPS 0.01 and at 2 and 4 h for both LPS 0.1 and LPS 1, but were not different from 0 h at any of the other time points (Figure 1).

### 3.2. Study 2: Local (GF-Pulp) and Systemic (Blood) Inflammatory Responses to Intradermal Pulp Injection of Various Dosages of Peptidoglycan (PGN)

**Figure 2:**
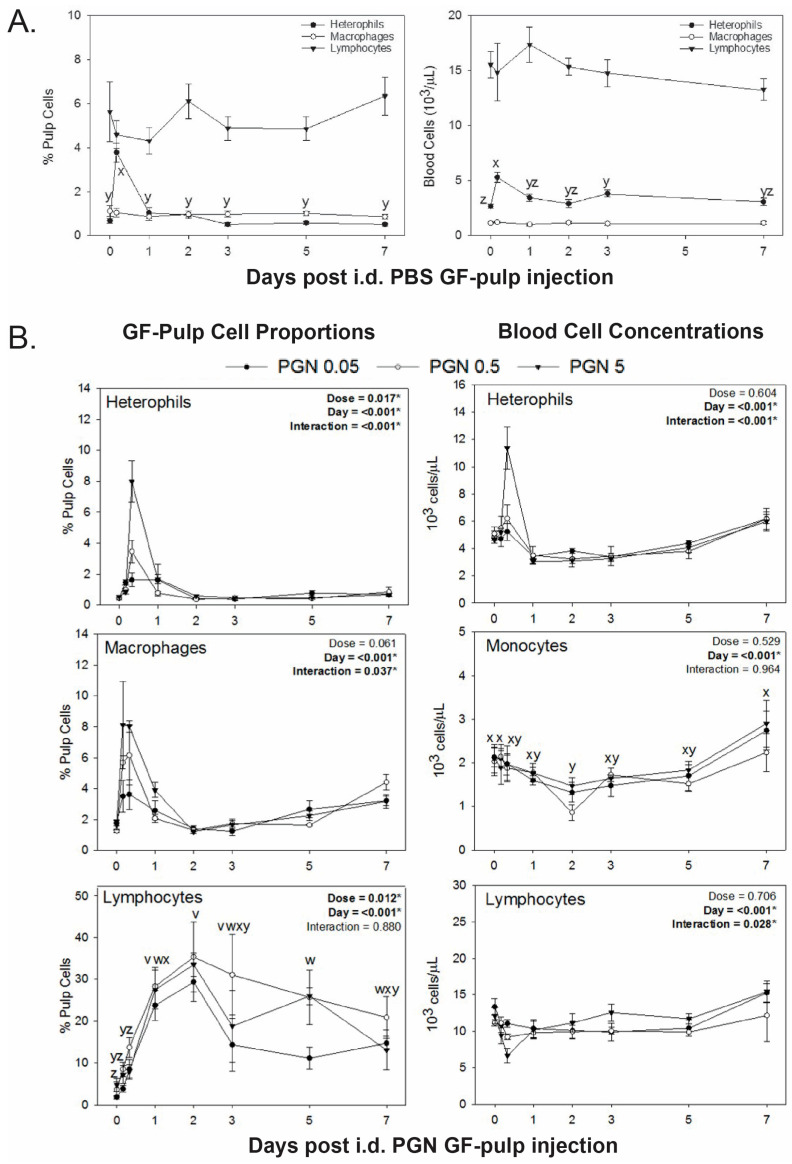
Changes in blood cell concentrations and pulp cell proportions of heterophils, monocytes/macrophages, and total lymphocytes after intradermal injection of (**A**) endotoxin-free PBS vehicle or (**B**) different doses of peptidoglycan into the pulp of growing feathers. Twenty growing feathers (GFs) of sixteen, 12-wk-old male and female chickens were injected with PBS (vehicle control), 0.05, 0.5, or 5 μg of peptidoglycan (PGN) per GF. Heparinized blood (0.5–1 mL) and one injected GF from each chicken were collected at 0 h (before injection), and at 4 h, 8 h, 1 d, 2 d, 3 d, 5 d, and 7 d post-injection for leukocyte population analysis. An automated hematology analyzer (Cell-Dyn) was used to determine heterophil, monocyte, and lymphocyte blood cell concentrations (10^3^ cells/μL). Pulp cell suspensions from GFs were prepared and immunofluorescently stained with a panel of fluorescence-conjugated mouse monoclonal antibodies to identify chicken macrophages and lymphocytes. Analysis of cell populations was conducted by flow cytometry and percentages of heterophils were based on size (FSC) and granularity (SSC) characteristics of leukocytes (CD45^+^). Data shown are means ± SEM; * indicates significant effects; n = 4 per time point and treatment group. Multiple means comparison of PGN dose effects in GFs revealed lower lymphocyte levels for PGN 0.05 on 5 d (*p* ≤ 0.05); v–z indicate day main effect differences; time points without a common letter are different.

Statistical analyses revealed treatment-by-time interactions for heterophil, monocyte/macrophage, and lymphocyte proportions (% pulp cells) in GF-pulp and their concentrations in the blood. As PBS appeared to result in different response profiles compared to PGN, treatment effects of PBS were analyzed separately for changes over time. Apart from a significant time effect for heterophil levels in GFs, i.e., elevated levels at 4 h (*p* ≤ 0.05), intradermal pulp injection of endotoxin-free PBS vehicle neither affected blood heterophil concentrations nor levels of monocytes/macrophages and lymphocytes in pulp and blood over the 7-day examination period (Figure 2A). Moreover, there were no changes in the levels of B cells and CD4-, CD8-, γδ TCR-, aβ1 TCR-, and αβ2 TCR-defined T cell subsets following pulp injection of endotoxin-free PBS (Table 2).

Two-way analysis of PGN treatments (PGN 0.05, 0.5, and 5) revealed treatment-by-time interactions for GF heterophil (*p* < 0.001) and macrophage (*p* = 0.037) infiltration and heterophil (*p* < 0.001) and lymphocyte (*p* = 0.028) concentrations in blood (Figure 2B). Injection of the lowest PGN dose (PGN 0.05), resulted in elevated heterophil infiltration at 8 h and 1 d, whereas for PGN 0.5 and PGN 5, heterophil levels were only elevated at 8 h. PGN 5 resulted in the highest heterophil infiltration. In the blood, heterophil concentrations were not affected by PGN treatments, except for elevated levels (*p* ≤ 0.05) at 8 h following intradermal pulp injection with PGN 5.

Independent of injection dose, PGN treatments resulted in elevated macrophage levels (*p* ≤ 0.05) in GF at 4, 8, and 24 h p.i., with maximal levels at 4 and 8 h p.i.; at 4, 8, and 24 h, macrophage levels in GFs were highest with PGN 5, followed by PGN 0.5 and PGN 0.05 (Figure 2B). Independent of PGN treatment, blood monocyte concentrations dropped slightly post-pulp injection, reaching lowest levels (*p* ≤ 0.05) at 2 d, and returning to pre-injection levels by 7 d (Figure 2B). 

Unlike LPS, pulp injection of PGN resulted in rapid, extensive, and sustained lymphocyte infiltration in GF-pulp. There was no treatment-by-time interaction (*p* = 0.880) for GF lymphocyte infiltration, but a main effect of time (*p* < 0.001) and a main effect of PGN dose (*p* = 0.012) (Figure 2B). Overall, lymphocyte levels in GF-pulps increased consistently post-PGN injection, reaching greatly elevated levels at 1 d and maximal levels at 2 d, then decreased from 5 to 7 d, but remained elevated throughout the 7-day study. There were no differences in lymphocyte levels between the PGN dosages, except for lower levels (*p* ≤ 0.05) with PGN 0.05 compared to PGN 0.5 and PGN 5 at 5 d. Two-way ANOVA of individual lymphocyte subpopulations revealed no treatment-by-time interactions (*p* ≥ 0.291) for any of the lymphocyte subpopulations examined (B cells and CD4-, CD8-, γδ TCR-, aβ1 TCR-, and αβ2 TCR-defined T cell subsets), no treatment effect on B cells and γδ T cells (*p* ≥ 0.05), but treatment main effects with *p* = 0.029, 0.039, 0.04, and 0.029 for CD4-, CD8-, aβ1 TCR-, and αβ2 TCR-defined T cell subsets, respectively. However, multiple means comparisons did not reveal significant differences between the treatment main effect means (*p* ≥ 0.05). For all lymphocyte subsets examined in PGN treated GFs, there was a significant (*p* < 0.01) main effect of time (Table 2). Multiple means comparisons between various time points showed that levels of B cells were elevated (*p* ≤ 0.05) by 1 d p.i., reached maximal levels at 2 d, remained near maximal levels through 5 d, and were still greatly elevated at 7 d (Table 2). Levels of γδ T cells were elevated by 4 h (*p* ≤ 0.05), peaked at 1 d, and decreased to levels intermediate to elevated levels at 4 h and 2 d, and those before PGN injections. All other T cell subsets (CD4-, CD8-, aβ1 TCR-, and αβ2 TCR-defined lymphocytes) were elevated by 1 d, reaching maximal levels at 1 to 2 d, then dropped to lower levels, but remained above pre-injection levels at 3 to 7 d (Table 2). For blood, there was no significant change in lymphocyte concentrations in response to PGN treatments, except for lower (*p* ≤ 0.05) lymphocyte levels at 8 h post i.d. pulp injection with the highest PGN dose (PGN 5) (Figure 2B).

### 3.3. Study 3: Local Leukocyte Infiltration and Cytokine Gene Expression in Response to Intradermal Pulp Injection of PBS, LPS, or PGN

#### 3.3.1. Leukocyte Infiltration

**Figure 3:**
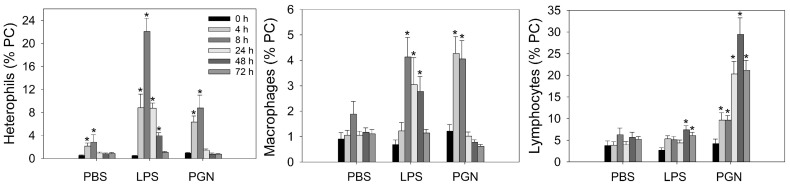
Proportions (% of pulp cells; PC) of heterophils, macrophages, and total lymphocytes in growing feather-pulps after intradermal pulp injection of endotoxin-free PBS vehicle, lipopolysaccharide (LPS), or peptidoglycan (PGN). Twenty growing feathers (GFs) of eighteen, 15-wk-old male chickens were injected with PBS (vehicle control), 1 μg of LPS or PGN per GF. One injected GF from each chicken was collected at 0 h (before injection) and at 4, 8, 24, 48, and 72 h post-injection for leukocyte population analysis. Pulp cell suspensions from GFs were prepared and immunofluorescently stained with a panel of fluorescence-conjugated mouse monoclonal antibodies to identify chicken macrophages and lymphocytes. Analysis of cell populations was conducted by flow cytometry, and percentages of heterophils were based on size (FSC) and granularity (SSC) characteristics of leukocytes (CD45^+^). Data shown are means ± SEM; n = 6 per time point and treatment group. * Indicates differences between pre- and post-injection means within a treatment group (*p* ≤ 0.05).

Two-way ANOVA revealed treatment-by-time interactions for the proportions (% pulp cells) of heterophils, macrophages, and lymphocytes, as well as for B cells and the various T lymphocyte subpopulations examined. Hence, treatment and time effects were analyzed within each time point and for each treatment, respectively. Injection of endotoxin-free PBS vehicle resulted in elevated levels (*p* ≤ 0.05) of heterophils at 4 and 8 h, while there was no change in levels of macrophages, lymphocytes, and lymphocyte subpopulations (Table 3) post vehicle injection (Figure 3, Table 3).

Injection of LPS resulted in greatly elevated (*p* ≤ 0.05) levels of heterophils from 4 h through to 48 h, reaching peak levels at 8 h. Levels of macrophages were elevated (*p* ≤ 0.05) at 8, 24, and 48 h, and those of lymphocytes at 48 h (Figure 3). However, levels of individual lymphocyte populations did not change (*p* ≥ 0.05) over time in response to LPS injection (Table 3).

Intradermal GF-pulp injection of PGN resulted in heterophil and macrophage infiltration at 4 and 8 h (*p* ≤ 0.05), returning to pre-injection levels by 24 h, whereas lymphocytes infiltration steadily increased starting at 4 h, reaching maximal levels at 48 h, and remained near maximal levels from 24 to 72 h (Figure 3). Analyses of individual lymphocyte populations revealed that levels of B cells were elevated (*p* ≤ 0.05) by 24 h, reached maximal levels at 48 h, and remained at this level at 72 h (Table 3). Levels of γδ T cells were elevated by 4 h (*p* ≤ 0.05) and remained at similar levels through 72 h. All other T cell subsets (CD4-, CD8-, aβ1 TCR-, and αβ2 TCR-defined lymphocytes), were elevated by 24 h, reaching peak levels at 48 h, and remained elevated at 72 h.

Treatment comparisons revealed that heterophil and macrophage infiltration were higher with LPS and PGN than PBS injection, whereby heterophil levels were highest with LPS and those of macrophages were similarly high with LPS and PGN (Figure 3). However, macrophage levels were elevated earlier (4 h vs. 8 h) but returned to baseline levels earlier (24 h vs. 72 h) with PGN than LPS, respectively. Infiltration of lymphocytes, and the various lymphocyte subpopulations examined, was highest in response to PGN injection and equally low in response to PBS and LPS administration (Figure 3, Table 3).

#### 3.3.2. Relative Cytokine mRNA Expression

Two-way ANOVA revealed treatment-by-time interactions (*p* < 0.01) for all cytokine genes examined, and there was no change in the relative expression levels for IL12A and IL21 with time or treatment (PBS, LPS or PGN). Hence, in the presence of treatment-by-time interactions, treatment and time effects were analyzed within each time point and for each treatment, respectively.

**Figure 4:**
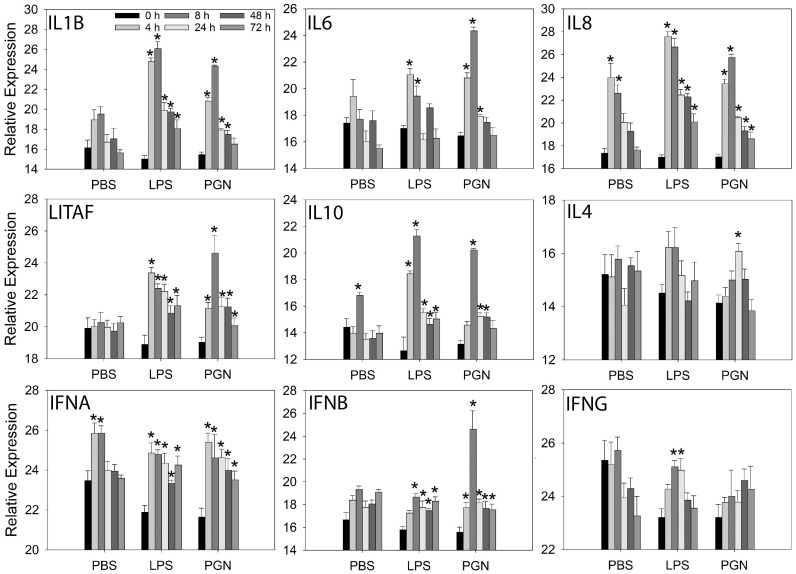
Relative cytokine gene expression in growing feather-pulps after intradermal pulp injection of endotoxin-free PBS vehicle, lipopolysaccharide (LPS), or peptidoglycan (PGN). Twenty growing feathers (GFs) of eighteen 15-wk-old male, egg-type chickens were injected with PBS (vehicle control), 1 μg of LPS, or 1 μg PGN per GF. One injected GF from each chicken was collected at 0 h (before injection) and at 4, 8, 24, 48, and 72 h post-injection for RNA isolation and relative cytokine mRNA expression analysis by RT-qPCR. Data shown are means ± SEM of relative mRNA expression (40 − ∆Ct); n = 6 per time point and treatment group. * Indicates differences between pre- and post-injection means within a treatment group (*p* ≤ 0.05).

Injection of endotoxin-free PBS vehicle, resulted in few cytokine gene expression changes, with elevated levels (*p* ≤ 0.05) observed only for IL8 at 4 and 8 h, IL10 at 8 h, and IFNA at 4 and 8 h (Figure 4).

Pulp injection of LPS or PGN affected the expression of most cytokines examined (Figure 4). For LPS, IL1B expression was greatly elevated from 4 to 72 h, with maximal levels at 4 and 8 h, whereas for PGN, IL1B expression was elevated from 4 to 48 h, reaching peak levels at 8 h that were similar in magnitude to those with LPS at 4 and 8 h. IL6 expression was elevated at 4 and 8 h in response to LPS and PGN injection, with similar levels at 4 h but higher levels with PGN than LPS at 8 h. For LPS, IL8 expression was greatly elevated from 4 to 72 h, with maximal levels at 4 and 8 h, while for PGN, IL8 was elevated from 4 to 48 h, reaching peak levels at 8 h that were similar in magnitude to those with LPS at 4 and 8 h. LITAF expression was elevated from 4 to 72 h for both treatments, with higher levels at 4 h with LPS compared to PGN. For LPS, IL10 expression was elevated from 4 to 72 h, reaching peak levels at 8 h, whereas for PGN, IL10 expression was elevated at 8, 24, and 48 h, reaching highest levels at 8 h that were not different from LPS peak levels. IL4 expression was not affected by LPS injection, but steadily increased with PGN injection to maximal levels at 24 h that were higher (*p* ≤ 0.05) than before injection. Expression profiles of IFNA were similar for LPS and PGN, with elevated levels from 4 to 72 h. Relative expression levels of IFNB were elevated at similar levels from 8 to 72 h with LPS, whereas for PGN, IFNB was elevated from 4 to 72 h, reaching peak levels at 8 h that were higher (*p* ≤ 0.05) than with LPS. IFNG expression levels were only affected by LPS, reaching maximal levels at 8 and 24 h, that were higher than before injection (Figure 4).

## 4. Discussion

To gain insights into temporal, qualitative, and quantitative aspects of the inflammatory responses generated by different bacterial MAMPs, we monitored leukocyte responses initiated by i.d. GF-pulp injection of LPS from *Salmonella* Typhimurium or PGN from *Staphylococcus aureus* at both the site of injection and in the peripheral blood. Using the GF-pulp cutaneous bioassay together with sampling of the peripheral blood, we were able to conduct longitudinal studies of the local (GF-pulp) and systemic (blood) leukocyte response profiles to these MAMPs in the same individuals. While this “two-window” approach was previously used to study the acute inflammatory response to LPS in broiler chickens [7], this is the first comprehensive study on the local and systemic inflammatory responses of PGN in the chicken model. Surprisingly, the two MAMPs generated divergent local inflammatory responses, i.e., for LPS, the response was dominated by heterophils and macrophages, and for PGN by a prolonged increase and presence of lymphocytes post i.d. GF-pulp injection. Below, we discuss the results for the two dose effect studies on LPS and PGN (Study 1 and 2, respectively) on leukocyte profiles at the site of injection and in the peripheral blood circulation and conduct side-by-side comparisons of results from Study 3 on the local inflammatory responses, including cytokine mRNA expression in GF-pulps following i.d. injection of PBS, LPS, or PGN.

### 4.1. Effect of Dose of LPS or PGN on the Local and Systemic Inflammatory Response Profiles in Egg-Type Chickens

#### 4.1.1. LPS

Observations made here using minimally invasive procedures to simultaneously monitor the acute inflammatory response at the site of LPS injection in GF-pulps and in the blood of egg-type chickens are in line with those in broiler chickens and confirm similarities between the avian and the mammalian inflammatory response initiated by LPS [3,7,9]. Independent of dose injected into GF-pulps, LPS recruited similar levels (% of pulp cells) of heterophils and monocytes/macrophages to the site of injection. Heterophils were the first type of leukocyte to appear and were accompanied and followed by monocyte/macrophage infiltration. For all LPS doses, heterophils remained the predominant phagocyte early in the LPS response (2–8 h p.i.), whereas macrophages remained near maximal infiltration levels at 8 and 24 h. This time course of inflammatory activity is also in agreement with the function of these phagocytes. As a first line of defense, heterophils are the first phagocytes to arrive in the inflamed tissue but are short-lived, whereas macrophages arrive more gradually, and their presence and activities are more sustained and diverse. Macrophages are considered the more professional phagocytes due to an extensive and diverse array of pattern recognition receptors, better ability to respond to and produce a variety of inflammatory mediators, as well as initiating tissue healing and restoring tissue homeostasis [3,20].

However, differences in leukocyte profiles in both the LPS injected GF-pulps and the blood were identified between the low LPS dose of 0.01 µg/GF (LPS 0.01) and the 10- and 100-fold higher doses (LPS 0.1 and LPS 1.0, respectively). Compared to LPS 0.01, pulp infiltration of heterophils and monocytes/macrophages in response to the higher LPS doses was delayed by 2 h and 4 to 8 h, respectively. Despite this delay, these leukocytes reached similar infiltration levels independent of dose through the remainder of the 24-h examination period examined.

The delay in heterophil infiltration with LPS 0.1 and LPS 1.0 was likely due to the drop in heterophil blood concentrations at 2 h p.i., which was not observed with LPS 0.01. Rather, with this low dose, heterophil concentrations in the blood were already elevated at 2 h, reaching maximal concentrations at 4 and 8 h, before returning to baseline concentrations at 24 h post GF-pulp injection. With the higher LPS 0.1 and LPS 1.0 doses, the initial drop in blood heterophils was followed by substantial increases, reaching levels similar to maximal levels of the low dose at 4 h, and continued to increase to peak levels at 8 h that were nearly double those of the low dose. By 24 h, heterophil concentrations returned to pre-injection levels with LPS 0.1, but remained elevated with LPS 1.0. It appears that the low LPS 0.01 dose resulted in a local inflammatory response where local activities signaled recruitment of heterophils from the blood into the affected tissue and stimulated more heterophil production and/or their release into the circulation. On the other hand, heterophil concentrations with the higher LPS 0.1 and 1.0 doses were like those described with intravenous (i.v.) injection of LPS in chickens [6], mice [4], and humans [5]. Specifically, Bowen et al. [6] reported a significant decrease in the peripheral blood concentration of heterophils 1 h post LPS i.v. injection (1 mg/kg body weight) which was followed by rapid and high increases (2.5- to 3.5-fold above baseline) in heterophil concentrations a few hours later. Hence, with the higher LPS doses, some of the injected LPS may have entered the blood along with the locally produced inflammatory mediators causing a response like that seen with i.v. injections. It is interesting that by 24 h heterophil concentrations were still elevated with LPS 1.0, but had returned to pre-injection levels with LPS 0.1. Whether this effect of the LPS 1.0 dose is due to greater inflammatory activity generated at the site of inflammation or more LPS entering the circulation, or both, needs further investigation.

While monocyte/macrophage infiltration into the pulp was observed with all LPS doses, monocyte concentrations in the blood dropped by 2 h with LPS 0.1 and 1.0, then returned to pre-injection levels by 4 to 8 h, but did not increase above pre-injection levels for any of the LPS doses. The drop in monocyte concentrations in the blood was still evident at the time macrophage levels in the injected tissue increased. Hence the drop and the lack of increased circulating monocyte concentrations may be explained by vascular adhesion of monocytes followed by gradual extravasation of monocytes into injected tissues.

For lymphocytes, the changes initiated by intradermal injection of LPS are opposite to those for heterophils, with both the proportions of lymphocytes in GF and lymphocyte concentrations in the blood dropping at 2 and 4 h following pulp injection of LPS. While levels of lymphocytes in the blood recovered by 8 to 24 h, normal lymphocyte levels in injected pulp were not restored by 24 h with the high LPS (LPS 0.1 and 1.0) doses. The overall decrease in total lymphocytes in the pulp of LPS injected GF could not be attributed to any one specific lymphocyte subpopulation, not even γδ T cells, which are known to have barrier- and innate-like functions. The drop in circulating lymphocytes with LPS administration has been repeatedly observed in both meat- and egg-type chickens [6,7,21]. The reason for the lower lymphocyte levels in blood and LPS injected pulp is not clear, but cytokines produced at sites of inflammation are known to stimulate expression of T and B cell chemokines in secondary lymphoid organs in mice and humans [3], and endotoxin is known to have indirect and direct toxic effects on lymphocytes [5,6,22].

It should be noted that 20 GFs were injected with LPS per chicken, hence, the overall dose of LPS was 0.2, 2, and 20 µg for the 0.01, 0.1 and 1.0 µg i.d. injections into the pulp of GFs. Compared to most mammals, chickens are known to tolerate high levels of LPS, where i.v. injection doses used typically ranged from 0.1 to 1 mg/kg body weight without mortality [23]. This study further emphasized that chickens are capable of effectively and appropriately responding to much lower LPS challenges, and hence, infections with Gram-negative bacteria.

#### 4.1.2. PGN

Information on the immunostimulatory effects of cell wall products from Gram^+^ bacteria is sparse, although Gram^+^ bacteria, such as *Mycobacteria*, are often included in adjuvants. The in vivo effects of PGN have not been established in poultry. The current study is the first to describe the nature of the inflammatory responses initiated by PGN in a complex tissue such as the dermis of GF-pulps and in the peripheral blood circulation over the course of 7 days following i.d. injection.

The in vivo effects of i.d. injected PGN were examined at three dosages ranging from 0.05 µg to 5 µg of PGN per GF. Considering this large range in PGN concentrations evaluated, it was surprising to find no or only minor differences in the leukocyte recruitment responses initiated by PGN, both in the injected pulp and in the peripheral blood. Independent of PGN dose, i.d. pulp injection resulted in increased levels of heterophils reaching peak levels at 8 h p.i. and returning to pre-injection levels by 24 h, in both the injected pulp and blood. Similar observations were reported for in vivo mouse studies examining leukocyte recruitment in lungs exposed to PGN [24]. Inhalation of PGN resulted in polymorphonuclear cells (assumed to be neutrophils) recruitment into the lungs that were eight times higher 4 h after than before PGN inhalation [24]. The heterophil infiltration into PGN injected GF-pulps was accompanied by increased levels of macrophages which returned to near pre-injection levels by 2 d. This influx of macrophages into the pulp was not reflected by increased monocyte levels in the blood; rather, monocyte concentrations dropped gradually following pulp injection, reaching significantly lower levels at 2 d, before gradually returning to basal levels by 7 d p.i.

The most striking observation in this PGN dose-response study was the rapid and substantial infiltration of lymphocytes, increasing more than 10-fold by 1 d, and their sustained presence at high levels throughout the 7-d study, independent of PGN dose. The increased proportion (% pulp cells) of lymphocytes in GFs was accompanied by a small drop in blood lymphocyte concentrations with the higher doses of PGN by 8 h p.i., but otherwise, blood lymphocyte concentrations were at normal levels throughout the 7-d study. The tremendous influx of lymphocytes within a day of PGN injection may explain this initial drop in blood lymphocytes concentrations. Intra-peritoneal injection of PGN was shown to increase expression of vascular adhesion molecules [25]. Hence, like with the higher doses of LPS, PGN may have entered the circulation, and the drop in the concentration of circulating lymphocytes may be due to vascular adhesion or redistribution to various tissues, rather than to extravasation into the 20 injected GFs alone. GF-pulp infiltrating lymphocytes included both B and T cells, as well as all T cell subpopulations examined, although the time-course of their pulp infiltration differed. γδ T cells levels were already elevated at 4 h, reaching peak levels at 1 d. Infiltration of γδ T cells was accompanied by αβ T cells that infiltrated more gradually over the first 8 h, but far exceeded those of γδ T cells by 1 d (approx. 10.7 vs. 3.6% of pulp cells, respectively) and remained at these peak levels at 2 d, before gradually dropping to still above baseline levels at 7 d. CD4^+^- and CD8^+^-T cell subsets followed the same trend as αβ T cells. While not examined here, it is possible that some of the CD8^+^ cells may have been γδ TCR^+^ or that some of the T cells were CD4^+^CD8^+^αβ TCR^+^ which are known to be present in chickens [26]. Infiltration of B cells followed a similar trend to those of T cells, with levels increasing 10-fold by 1 d but then, unlike T cells, continued to increase at 2 d to mean peak levels of 17.2%; they remained at this level through 5 d before dropping to still greatly elevated levels at 7 d. Overall, from 2 d onward B cells levels were higher than those of T cells.

Unfortunately, there are no comparable in vivo studies on the dose responses to PGN nor on local and systemic inflammatory responses initiated in vivo by i.d. PGN injection. We further discuss the divergent inflammatory responses to LPS and PGN at the site of injection with the leukocyte profile and cytokine mRNA expression results from Study 3.

#### 4.1.3. Vehicle

In all three studies reported here, injection controls were included in the form of endotoxin-free PBS (PBS) vehicle administered by i.d. GF-pulp injection at the same volume (10 μL/GF) as the various doses of LPS or PGN. In all three studies, there were slight increases in heterophils and macrophages in injected pulps, which were nearly or marginally significant early (4–8 h p.i.) in the response and returned to pre-injection levels by 1 d. In the blood, only heterophil concentrations increased, also early (4–8 h) post i.d. GF-pulp injection of PBS. Examination of local cytokine mRNA expression in Study 3 post-PBS injection revealed increases in interleukin (IL-8) and interferon-alpha (IFN-α) mRNA at 4 and 8 h p.i., and IL-10 at 8 h, which concurs with elevated presence of heterophils and macrophages. The observed cell recruitment and cytokine expression profiles are likely due to tissue injury caused by the injection and an effort to initiate tissue repair. In this context, the numerical increases in IL-1β and IL-6 mRNA at 4–8 h p.i. may also be of biological relevance [3,7].

### 4.2. Concurrent Study of Local Activities Initiated by i.d. GF-Pulp Injection of LPS or PGN Confirms Their Divergent Inflammatory Response Profiles

In Study 3, the local immune activity initiated in response to i.d. GF-pulp injection of LPS or PGN was examined concurrently at a dose known to produce a reliably strong response for each of the MAMPs, both in chickens and mammals. The 72-h examination period was chosen to cover the time-course when the temporal, quantitative, and qualitative aspects of their local inflammatory responses were most evident. Limited gene expression of cytokines at the transcriptome level was included to better understand activities in the inflamed GF-pulps.

As expected, both LPS and PGN injection into the pulp of GFs resulted in recruitment of heterophils, with LPS stimulating a more than 2-fold higher increase in heterophils than PGN by 8 h p.i. With LPS, heterophil levels gradually declined returning to pre-injection levels by 72 h, whereas with PGN, baseline levels were already observed by 24 h. In response to LPS, macrophages reached maximal levels by 8 h and remained elevated by 48 h, returning to baseline levels by 72 h, whereas with PGN, macrophages reached maximal levels similar to those with LPS at 4 and 8 h but returned to baseline levels by 24 h p.i. Confirming our observations in the PGN dose response study, only PGN resulted in high recruitment and sustained presence of lymphocytes over the three-day period in PGN injected GFs. As before, γδ T cells were already elevated by 4 h, while levels of other T cell subpopulations (CD4^+^, CD8^+^, αβ1-, and αβ2-T cells) and B cells were elevated by 24 h, reaching peak levels at 48 h. By 72 h, levels of all T cell populations started to decline, whereas those of B cells remained at peak levels.

The leukocyte profiles in GF-pulps following intradermal injection of LPS agree with the local, acute, 24-h inflammatory response profiles observed in i.d. injected GF-pulps in broilers and the dose-response study for egg-type chickens reported here, as well as with other in vivo and in vitro studies in chickens and mammals. Santamaria et al. [27] recently reported a 72-h longitudinal study on the local GF-pulp inflammatory responses to killed autologous *Salmonella* vaccines or LPS from *Salmonella* Enteriditis administered in a water–oil–water emulsion vehicle in egg-type pullets. They observed similar heterophil- and macrophage-dominated response profiles for both the vaccines and LPS, although levels of both types of phagocytes dropped more gradually and remained elevated throughout the 72-h study. From this study, it appeared that the local leukocyte profiles in response to the vaccine could be attributed primarily to LPS, and the prolonged phagocyte presence to the emulsion vehicle [27].

For PGN, there are no comparative studies to the longitudinal in vivo study on the local tissue/cellular response described here. However, in a study whereby killed *Mycobacterium butyricum*, a Gram-positive bacterium, was injected into the dermis of wattles, wing webs, or GF-pulps revealed similar qualitative, quantitative, and temporal characteristics of the leukocyte response profiles as those observed here for PGN [8]. The only difference between the responses to *M. butyricum* and PGN was a prolonged and heightened macrophage presence during the latter part (2 to 7 d) in *M. butyricum* injected GF-pulps. This difference may be attributable, in part, to the prior sensitization of chickens with *M. butyricum* in this study before injecting *M. butyricum* into GF-pulps and other skin derivatives [8].

PGN is thought to stimulate leukocytes primarily by interaction with TLR 2, in both avian and mammalian species [13,14,28]. In mammals and chickens, TLR 2 was shown to be expressed on B and T lymphocytes [13,28] and studies with Freund’s complete adjuvant (of which PGN from *Mycobacteria* is a major immunostimulatory component) show recruitment of lymphocytes to the injection site [29]. Considering the rapid response time by T and B lymphocytes in the current study, it is likely that the recruitment of lymphocytes with PGN was not antigen-specific, as adaptive immune responses typically take days to weeks for antigen-specific effector cells to be produced [3]. Rather, the lymphocyte recruitment and possible local activation and proliferation in injected GFs is likely mediated by TLRs present on both types of lymphocytes [3,13,28]. In vitro studies, using human peripheral blood mononuclear cell cultures, have shown PGN to function as a T and B cell mitogen and polyclonal activator, whereby B cell proliferation required the presence of T cells, but polyclonal B cell activation and differentiation was relatively T cell independent [30]. TLR2 has also been shown to be present on chicken T and B cells [28], hence the increasing presence of lymphocytes may, in part, be due to local proliferation of recruited lymphocytes. More research is needed to determine the mechanism of lymphocyte recruitment and retention in response to i.d. injection of PGN. It is likely that recruitment of lymphocytes is mediated by PGN stimulated chemokines produced by resident and recruited leukocytes rather than a direct effect on T and B cells.

To gain insight into gene expression in inflamed tissue following LPS or PGN injection, we conducted RT-qPCR to determine mRNA expression of key inflammatory and regulatory cytokines in GF-pulps; specifically, IL-1β, IL-4, IL-6, IL-8 (aka CXCL8), IL-10, IL-12, IL-21, IFN-α, -β, and -γ, as well as LITAF, a transcription factor for TNF-α expression. The observation of elevated expression of proinflammatory cytokine genes IL1β and IL6, LITAF, and chemoattractant IL8 (CXCL8) in the pulp of GFs early (4–8 h) after injection with LPS or PGN highlights the inflammatory nature of LPS and PGN. Moreover, these results agree with the early infiltration of heterophils and macrophages into the injected GF-pulps observed in the cellular analyses. Similar transcriptional increases in inflammatory gene expression have been observed in studies with whole microbe or MAMP stimulation of individual heterophil and macrophage populations [11,14,15,31]. The increased transcriptional expression of IL10, from 4 to 72 h and 8 to 48 h with LPS and PGN, respectively, may indicate an attempt to regulate the inflammatory response, as IL-10 is known to downregulate macrophage co-receptors as well as block inflammatory pathways [3].

Additionally, both LPS and PGN stimulated the expression of Type 1 interferons (IFN-α and β), whereas only LPS resulted in increased expression of IFNG. As IFN-γ is a major activator of macrophages [20], this observation agrees with the sustained macrophage presence from 8 to 48 h with LPS, which was only observed at 4–8 h with PGN. However, IFN-γ is typically produced by type 1 T helper (Th1) effector cells that are likely not present in this innate LPS response, especially as levels of CD4^+^ lymphocytes did not change in response to LPS. It is, however, possible that the numerically elevated levels of γδ T cells and perhaps natural killer cells and other innate lymphoid cells present in the tissue, may be the source of the increased IFNG expression in the LPS injected GF-pulps. On the other hand, considering the extensive T cell presence in PGN injected pulps, it is surprising that IFNG expression was not observed with PGN. This supports the idea that recruited T cells are likely naïve and not cytokine producing effector cells. Hence, it is also likely that the increased expression of IL4 observed with PGN may be due to IL-4 produced by activated mast cells in the inflamed tissue rather than by Th2 effector cells. Lastly, the higher IL6 expression, together with IL4 in PGN injected pulps, may be driving the increased and sustained presence of B cells from 24 h onward. Overall, the targeted RT-qPCR analyses conducted did not reveal functional activities in the injected pulps that could explain the lymphocyte-dominated inflammatory response with PGN. Further research is needed to address the mechanisms underlying the divergent responses initiated by LPS and PGN in vivo.

## 5. Conclusions

Intradermal administration of LPS or PGN revealed temporal, qualitative, and quantitative differences in the local inflammatory responses to these MAMPs. Specifically, i.d. GF-pulp injection of LPS resulted in a heterophil and monocyte/macrophage infiltration, whereas PGN initiated a prolonged lymphocyte-dominated response at the site of injection. For LPS, both the local (GF-pulp) and systemic (blood) leukocyte profiles were altered by the i.d. injection. For PGN, however, the abundance of lymphocyte presence in injected GF-pulps was not associated with similar concurrent changes in the concentrations of circulating lymphocytes. Hence, examination of leukocyte profiles in the peripheral blood does not necessarily reflect inflammatory activities taking place at the site of infection. Limited cytokine gene expression analyses of injected GF-pulp could not explain the divergent inflammatory response profiles with LPS compared to PGN. Considering *Mycoplasma butyricum*, a PGN-containing Gram^+^ bacterium, has been used as an indicator of cell-mediated immune capability in chickens [32,33], it is possible that PGN is the main component that directs the development of cellular immunity. More research is needed to identify the mechanism underlying the nature of the local inflammatory response initiated by PGN, including examination of TLR and cytosolic PRR expression and signaling pathways, local vascular activities such as selectin and integrin expression on endothelial cells of venules, secretion of chemokines by sentinel/recruited cells, as well as proliferative activity of the recruited lymphocytes. The minimally invasive two-window approach offers the opportunity to dissect local (GF-pulp) and systemic (blood) inflammatory responses to MAMPs in vivo, from initiation to resolution. Knowledge gained from these studies may find direct application in the identification of chickens with robust innate immune system defenses.

## Figures and Tables

**Table 1:** Primer and probe sequences ^1^ for target genes.

Target	Primers and Probe	Sequences (5′to 3′)	Accession NO.
28S	Forward	GGCGAAGCCAGAGGAAACT	X59733
	Reverse	GACGACCGATTTGCACGTC	
	Probe	(FAM)-AGGACCGCTACGGACCTCCACCA-(TAMRA)	
IL1B	Forward	GCTCTACATGTCGTGTGTGATGAG	AJ245728
	Reverse	TGTCGATGTCCCGCATGA	
	Probe	(FAM)-CCACACTGCAGCTGGAGGAAGCC-(TAMRA)	
IL4	Forward	AACATGCGTCAGCTCCTGAAT	AJ621735
	Reverse	TCTGCTAGGAACTTCTCCATTGAA	
	Probe	(FAM)-AGCAGCACCTCCCTCAAGGCACC-(TAMRA)	
IL6	Forward	GCTCGCCGGCTTCGA	AJ250838
	Reverse	GGTAGGTCTGAAAGGCGAACAG	
	Probe	(FAM)-AGGAGAAATGCCTGACGAAGCTCTCCA-(TAMRA)	
IL8 (CXCL8)	Forward	GCCCTCCTCCTGGTTTCA	AJ009800
	Reverse	TGGCACCGCAGCTCATT	
	Probe	(FAM)-TCTTTACCAGCGTCCTACCTTGCGACA-(TAMRA)	
IL10	Forward	CATGCTGCTGGGCCTGAA	AJ621614
	Reverse	CGTCTCCTTGATCTGCTTGATG	
	Probe	(FAM)-CGACGATGCGGCGCTGTCA-(TAMRA)	
IL12A	Forward	TGGCCAAGGGACTCAACTG	NM_213588.1
	Reverse	ACCTCTTCAAGGGTGCACTCA	
	Probe	(FAM)-CCGCTGCAAACGAGGCACTCCT-(TAMRA)	
IL21	Forward	GTGGTGAAAGATAAGGATGTCGAA	NM_001024835.1
	Reverse	TGCCATTCTGGAAGCAGGTT	
	Probe	(FAM)-TGCTGCATACACCAGAAAACCCTGGG-(TAMRA)	
IFNA	Forward	GACAGCCAACGCCAAAGC	U07868
	Reverse	GTCGCTGCTGTCCAAGCATT	
	Probe	(FAM)-CTCAACCGGATCCACCGCTACACC-(TAMRA)	
IFNB	Forward	CCTCCAACACCTCTTCAACATG	X92479
	Reverse	TGGCGTGTGCGGTCAAT	
	Probe	(FAM)-TTAGCAGCCCACACACTCCAAAACACTG-(TAMRA)	
IFNG	Forward	GTGAAGAAGGTGAAAGATATCATGGA	Y07922
	Reverse	GCTTTGCGCTGGATTCTCA	
	Probe	(FAM)-TGGCCAAGCTCCCGATGAACGA-(TAMRA)	
LITAF	Forward	AAGACAAAATTTGCAGGCTGTTT	AB058634
	Reverse	GGAGCAGACATGATATATGACTGAAATAA	
	Probe	(FAM)-TGCCTCTGCCATCAGCTCTTTTGTGC-(TAMRA)	

^1^ Primer and probe oligos were synthesized by Eurofins MWG Operon LLC, Huntsville, AL, USA.

**Table 2:** B cells and T cell subset profiles in pulp of growing feathers following intradermal pulp injection of endotoxin-free phosphate-buffered saline (PBS) or peptidoglycan (PGN) in 12-week-old egg-type chickens ^1^.

Lymphocyte	Treatment	0 h	4 h	8 h	1 d	2 d	3 d	5 d	7d	*p* (Time)
Bu-1^+^	PBS	0.93 ^2^	0.66	0.75	0.75	1.07	0.57	0.86	0.97	0.305
	PGN	1.12 ^3^ z	1.26 z	2.98 z	11.7 xy	17.2 x	12.0 xy	13.5 xy	9.03 y	<0.001
CD4^+^	PBS	1.79	1.12	1.02	1.00	1.45	0.97	1.09	1.64	0.365
	PGN	1.26 z	2.47 zy	3.65 zy	9.04 xw	10.0 w	6.26 xy	5.84 y	4.79 y	<0.001
CD8^+^	PBS	2.52	2.01	2.12	2.42	3.28	2.94	3.07	3.33	0.102
	PGN	1.83 z	1.90 z	2.83 z	7.98 w	7.18 wx	4.60 xy	4.98 xy	3.95 y	<0.001
γδ TCR^+^	PBS	1.20	1.52	1.37	1.10	1.42	1.13	1.00	1.36	0.363
	PGN	0.96 z	2.29 y	2.86 xy	3.61 x	2.48 y	1.38 yz	1.33 yz	1.36 yz	<0.001
αβ1 TCR^+^	PBS	2.67	1.79	1.97	1.73	2.65	2.30	2.15	2.87	0.149
	PGN	1.90 z	2.01 yz	2.87 yz	7.51 w	6.75 wx	4.35 xy	3.99 y	3.92 y	<0.001
αβ2 TCR^+^	PBS	0.81	0.61	0.67	0.70	0.95	0.84	0.83	1.17	0.148
	PGN	0.34 z	0.812 z	1.19 yz	3.12 wx	3.76 w	2.36 xy	1.84 yz	1.42 y	<0.001

^1^ The pulp of 20 growing feathers (GFs) of 12-wk-old male and female egg-type chickens was intradermally injected with PBS or 0.05, 0.5, or 5 μg of PGN per GF; four chickens/treatment; 10 μL/GF. For each chicken, one GF collected before injection (0 h) and at 4 h, 8 h, and 1, 2, 3, 5, and 7 d post-injection was used to prepare pulp cell suspensions for direct immunofluorescent staining and cell population analyses by flow cytometry. B cells (Bu-1^+^) and CD4-, CD8-, and T cell receptor (TCR)-defined T cell subsets in the cell suspension were identified using a panel of chicken lymphocyte-specific fluorescently labeled mouse monoclonal antibodies. ^2^ Mean lymphocyte population (% pulp cells), n = 4 per time point and treatment group. ^3^ For PGN, there were no time-by-dosage interactions (*p* > 0.05). Hence, main time effect means across dosage are shown (% pulp cells; n = 12). w–z indicates time differences; within a treatment, time points without a common letter are different.

**Table 3:** B cells and T cell subsets in pulp of growing feathers following intradermal pulp injection of endotoxin-free phosphate buffered saline (PBS), lipopolysaccharide (LPS), or peptidoglycan (PGN) in male, egg-type chickens ^1^.

Cell-Type	Treat	0 h	4 h	8 h	24 h	48 h	72 h
Bu-1^+^	PBS	0.58 ± 0.20 ^2^	0.65 ± 0.13	1.34 ± 0.38	0.70 ± 0.14 b	0.92 ± 0.19 b	0.67 ± 0.09 b
	LPS	0.44 ± 0.11	1.04 ± 0.23	1.34 ± 0.19	1.46 ± 0.20 b	1.75 ± 0.17 b	1.11 ± 0.30 b
	PGN	1.12 ± 0.25 z	1.07 ± 0.08 z	1.60 ± 0.10 z	6.50 ± 1.08 a,y	10.8 ± 1.96 a,x	9.33 ± 0.97 a,x
CD4^+^	PBS	0.64 ± 0.23	0.51 ± 0.10	1.21 ± 0.55	0.51 ± 0.10 b	0.76 ± 0.26 b	0.81 ± 0.11 b
	LPS	0.57 ± 0.21	1.07 ± 0.29	1.76 ± 0.40	0.99 ± 0.22 b	2.04 ± 0.74 b	1.44 ± 0.17 b
	PGN	0.77 ± 0.13 z	2.47 ± 0.29 z	1.75 ± 0.28 z	6.18 ± 1.63 a,xy	8.28 ± 2.00 a,x	5.58 ± 0.82 a,y
CD8^+^	PBS	2.15 ± 0.67	2.37 ± 0.61	3.99 ± 1.16	2.68 ± 0.48 b	3.64 ± 0.61 b	4.09 ± 0.49
	LPS	1.28 ± 0.56	2.41 ± 0.39	2.82 ± 0.56	1.68 ± 0.32 b	4.06 ± 0.32 b	3.28 ± 0.50
	PGN	3.37 ± 0.60 z	3.63 ± 0.48 z	4.04 ± 0.48 z	5.03 ± 1.39 a,yz	8.11 ± 1.19 a,y	4.84 ± 0.88 yz
γδ TCR^+^	PBS	0.69 ± 0.22	1.11 ± 0.36 b	1.32 ± 0.42 b	0.83 ± 0.28 b	1.25 ± 0.45 b	1.10 ± 0.21 b
	LPS	0.58 ± 0.14	1.44 ± 0.27 b	1.36 ± 0.36 b	0.99 ± 0.24 b	1.33 ± 0.15 b	1.35 ± 0.13 b
	PGN	1.36 ± 0.28 z	3.57 ± 1.01 a,y	3.31 ± 0.93 a,y	3.57 ± 0.49 a,y	3.74 ± 0.32 a,y	3.04 ± 0.42 a,y
αβ_1_ TCR^+^	PBS	1.78 ± 0.45	1.42 ± 0.34	2.49 ± 0.70	1.65 ± 0.32 b	2.35 ± 0.53 b	2.38 ± 0.29 b
	LPS	1.20 ± 0.32	2.13 ± 0.32	1.71 ± 0.35	1.43 ± 0.34 b	3.23 ± 0.56 b	2.58 ± 0.24 b
	PGN	2.66 ± 0.53 z	3.64 ± 0.51 z	3.45 ± 0.43 z	7.25 ± 1.37 a,y	10.5 ± 1.63 a,x	6.46 ± 0.98 a,y
αβ_2_ TCR^+^	PBS	0.71 ± 0.23	0.67 ± 0.15	1.07 ± 0.22	0.77 ± 0.16 b	1.12 ± 0.23 b	1.02 ± 0.15 b
	LPS	0.48 ± 0.18	0.71 ± 0.07	0.69 ± 0.13	0.51 ± 0.10 b	1.06 ± 0.13 b	1.04 ± 0.17 b
	PGN	1.06 ± 0.18 z	1.34 ± 0.21 z	1.28 ± 0.15 z	2.82 ± 0.52 a,y	4.15 ± 0.68 a,x	2.24 ± 0.21 a,y

^1^ The pulp of 20 growing feathers (GFs) of 15-wk-old male, egg-type chickens were intradermally injected with PBS or 1 μg of LPS or PGN; six chickens/treatment; 10 μL/GF. For each chicken, one GF collected before injection (0 h) and at 4, 8, 24, 48, and 72 h post-injection was used to prepare pulp cell suspensions for direct immunofluorescent staining and cell population analyses by flow cytometry. B cells (Bu-1^+^) and CD4-, CD8-, and T cell receptor (TCR)-defined T cell subsets in the cell suspensions were identified using a panel of chicken lymphocyte-specific fluorescently labeled mouse monoclonal antibodies. ^2^ Mean lymphocyte population (% pulp cells) ± SEM, n = 6. a,b indicates treatment differences; within a time point, treatments without a common letter are different (*p* ≤ 0.05). x–z indicates time differences; within a treatment, time points without a common letter are different (*p* ≤ 0.05).

## Data Availability

The data presented in this study are available on request from the corresponding author.
